# Hepatotoxicity of Small Molecule Protein Kinase Inhibitors for Cancer

**DOI:** 10.3390/cancers15061766

**Published:** 2023-03-14

**Authors:** Mauro Viganò, Marta La Milia, Maria Vittoria Grassini, Nicola Pugliese, Massimo De Giorgio, Stefano Fagiuoli

**Affiliations:** 1 Gastroenterology Hepatology and Transplantation Unit, ASST Papa Giovanni XXIII, 24127 Bergamo, Italy; 2 Section of Gastroenterology & Hepatology, Department of Health Promotion Sciences Maternal and Infant Care, Internal Medicine and Medical Specialties, PROMISE, University of Palermo, 90127 Palermo, Italy; 3 Department of Gastroenterology, Division of Internal Medicine and Hepatology, IRCCS Humanitas Research Hospital, 20089 Rozzano, Italy; 4 Gastroenterology, Department of Medicine, University of Milan Bicocca, 20126 Milan, Italy

**Keywords:** cancer, drug-induced liver injury, hepatitis, hepatotoxicity, liver failure, tyrosine kinase inhibitors

## Abstract

**Simple Summary:**

This review reports the risk and management of the hepatotoxicity of all the approved protein kinase inhibitors (PKIs) for cancer. Hepatotoxicity is one of the major safety concerns of these drugs, as reflected by the discontinuation of the development of some of them due to liver injury, or by the significant number of warnings for hepatotoxicity reported in drug labeling. Although these side effects are usually reversible by dose adjustment or therapy suspension, or by switching to an alternative PKI, and fatality is uncommon, all patients undergoing these drugs should be carefully pre-evaluated and monitored during treatment.

**Abstract:**

Small molecule protein kinase inhibitors (PKIs) have become an effective strategy for cancer patients. However, hepatotoxicity is a major safety concern of these drugs, since the majority are reported to increase transaminases, and few of them (Idelalisib, Lapatinib, Pazopanib, Pexidartinib, Ponatinib, Regorafenib, Sunitinib) have a boxed label warning. The exact rate of PKI-induced hepatoxicity is not well defined due to the fact that the majority of data arise from pre-registration or registration trials on fairly selected patients, and the post-marketing data are often based only on the most severe described cases, whereas most real practice studies do not include drug-related hepatotoxicity as an end point. Although these side effects are usually reversible by dose adjustment or therapy suspension, or by switching to an alternative PKI, and fatality is uncommon, all patients undergoing PKIs should be carefully pre-evaluated and monitored. The management of this complication requires an individually tailored reappraisal of the risk/benefit ratio, especially in patients who are responding to therapy. This review reports the currently available data on the risk and management of hepatotoxicity of all the approved PKIs.

## 1. Introduction

**Figure 1:**
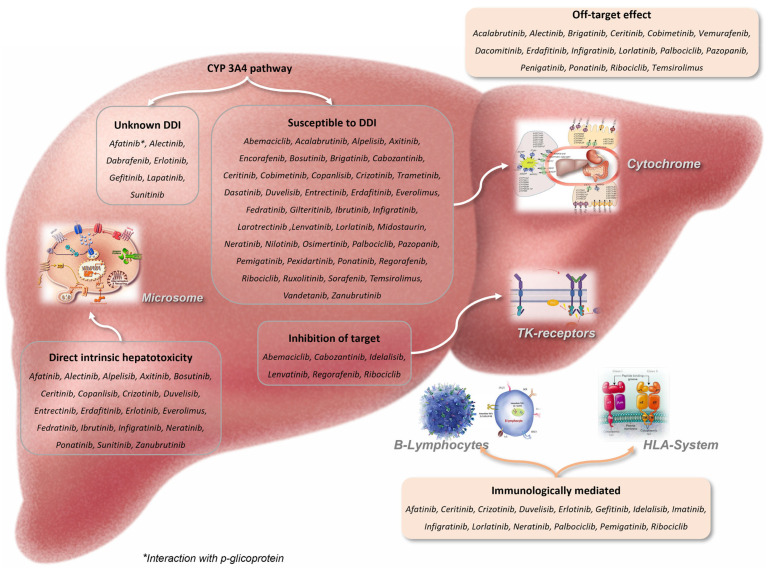
The different pathways of liver injury of the PKIs approved for cancer.

The phosphorylation of proteins by protein kinases is an important activating mechanism in the communication of signals within a cell and the regulation of both cellular activity and function [1,2]. These kinases play a major role in the cellular biochemical pathways involved in the transduction of extracellular signals regulating cellular responses including differentiation, proliferation, survival apoptosis, protein synthesis and other metabolic aspects of the cell cycle. Each kinase functions as an “on” switch, but they can also be overexpressed, become mutated or get blocked in the “on” position, resulting in the unregulated growth of the cell. Therefore, every overexpression, dysregulation or interference of these proteins’ activity may cause metabolic, autoimmune or inflammatory disorders, or lead to cancer [3]. For these reasons, the suppression of aberrantly expressed kinases has become an attractive strategy for patients with cancer. After Imatinib, the first U.S. Food and Drug Administration (FDA) approved small molecule tyrosine kinase inhibitor (TKI) in 2001, many others have been approved, and as of now there are 62 PKIs approved for cancer treatment. While these agents are generally well tolerated, their association with serious adverse events on a number of different organs, including the liver, have been reported. The liver injury secondary to PKIs involves:

- The production of several potentially toxic or immunogenic intermediates occurring within the CYP 3A4 pathway, that might alter both endogenous metabolism and cellular function leading to organ damage;
- The inhibition of cellular kinases or the “off-target effect” due to the production of toxic metabolites;
- The intrinsic hepatotoxicity by direct activity against essential intracellular kinases in hepatocytes;
- The immunologically mediated effect of the accumulation of immunogenic intermediates or B cell-driven autoimmunity (Figure 1).

However, the rates of confirmed PKI-hepatotoxicity remain poorly defined because the majority of data comes from pre-registration or registration trials on highly selected patients, and the comprehensive safety information coming from clinical practice is often missing as a result of the lack of a routinely planned on-treatment determination of aminotransferase levels. Therefore, only the most striking adverse events, leading to liver injury, are eventually published as case reports. The real risk for PKI-related severe hepatotoxicity in cancer patients remains an open issue, particularly due to the fact that PKIs are frequently utilized either sequentially or in combination with other chemotherapies. These other chemotherapies can be hepatotoxic in individuals with advanced cancers whose immune system or liver regeneration capacity may be hampered by an already impaired liver function, either due to preexisting conditions or therapies, or by malignant involvement of the liver. It must be noted that causality assessment methods remain controversial because some PKIs were marketed for a relatively short period of time, exposing only a small number of patients and therefore preventing the identification of idiosyncratic hepatotoxicity. Despite the fact that the majority of PKIs is reported to cause an increase in transaminases, and some of them have warnings for liver injury in regulatory documents, they are not at the top of the list of drugs that most frequently can cause hepatotoxicity [4,5,6,7,8,9,10]. Moreover, some PKIs may induce a hepatitis B virus reactivation (HBVr) either directly, by acting as an immunosuppressant, or indirectly, after a tumor response, supporting the hypothesis of a relation with immune restoration. In vitro studies have indeed reported that such therapies are capable of inhibiting T cell activation and proliferation, thus restoring the function of plasmacytoid dendritic cells, the well-known crucial effectors of innate immunity [11,12]. The rate of HBVr in oncologic HBsAg positive patients treated with PKI without antiviral prophylaxis is nearly 10% [13,14,15,16,17,18,19,20]. For this reason, the patients who would be the best candidates to receive these drugs should be screened for serologic markers of HBV infection, including hepatitis B surface antigen (HBsAg) and anti-hepatitis B core antigen (anti-HBc). HBsAg-positive patients should receive prophylaxis against HBVr with an oral antiviral agent, whereas patients with resolved infection, i.e., HBsAg negative/anti-HBc positive carriers with or without antibodies against HBsAg (anti-HBs), should be monitored with serum HBV DNA and/or HBsAg every three months, with a prompt initiation of an antiviral agent before the clinical onset of HBVr-related liver injury [21].

A systematic search on the PubMed/MEDLINE and LiverTox database of the data on PKI-hepatotoxicity using the PKI names and ‘liver’, ‘hepatotoxicity’, ‘hepatitis’, ‘drug induced liver injury’, ‘liver failure’ and ‘hepatic failure’ was carried out, and the resulting records were reviewed to collect relevant information in combination with those available on drug labeling for all the approved PKIs for cancer.

## 2. Relevant Section

In Table 1, the primary target, indications and rate of any alanine aminotransferase (ALT) increase, the Grade 3/4 hepatotoxicity according to the Common Terminology Criteria for Adverse Events (CTCAE) is reported for each PKI: Grade 1 [ALT > 3 × upper limit of normal (ULN)]; Grade 2 (ALT > 3 − 5 × ULN) without increase in total bilirubin > 2 × ULN; Grade 3 (ALT > 5 − 20 × ULN) without increase in total bilirubin > 2 × ULN; Grade 4 (ALT > 20 × ULN), and the drug labeling warnings for liver injury or boxed warnings for liver injury are taken into account.

### 2.1. Abemaciclib

ALT elevations were not reported during the prelicensure study [22], whereas phase II/III trials showed a moderate rate (30–48%) of any ALT elevation (G3/4: 4.6–7%) without reported cases of liver failure [23,24,25,26]. The median time to onset of G3/4 ALT increase ranged from 57 to 87 days, and the median time to resolution to <G3 was 14 days. Since the approval and more widescale use of Abemaciclib, there have been no published reports of its hepatotoxicity. Liver injury tests (LITs), i.e., ALT, AST and serum bilirubin, should be performed before starting treatment and every two weeks for the first two months, monthly for the next two months and then as clinically indicated. For G1/2 hepatotoxicity, no dose modification is required; with persistent or recurrent G2/3 hepatotoxicity, the dose should be suspended until toxicity resolves to baseline, or G1 and drug may be resumed at the next lower dose. The elevation of aminotransferase >3 × ULN with total bilirubin >2 × ULN, in the absence of cholestasis or G4 hepatotoxicity, requires Abemaciclib discontinuation. There is no information regarding cross-reactivity and the risk for hepatic injury between Abemaciclib and the other cyclin-dependent kinase (CDK) inhibitors, Ribociclib or Palbociclib, for breast cancers [27]. No dosage adjustments are necessary in patients with mild or moderate hepatic impairment (Child-Pugh A or B). The dosing frequency should be reduced to 150 mg once daily if Abemaciclib is given in combination with fulvestrant, tamoxifen or an aromatase inhibitor, or 200 mg once daily as monotherapy in patients with severe hepatic impairment (Child-Pugh C).

### 2.2. Acalabrutinib

There were no cases of ALT increase during prelicensure study [28], whereas phase II/III trials reported a mild rate (4.5–20%) of ALT elevations (G3/4: 1–1.9%) in monotherapy, which significantly increased when the drug was used in combination with Obinutuzumab (30% and 7%, respectively), however without cases of liver failure [29,30,31,32,33,34]. This drug has been associated with cases of HBVr. Acalabrutinib should be avoided in patients with severe hepatic impairment (Child-Pugh C), whereas no dosage adjustment is recommended in patients with mild to moderate hepatic impairment (Child-Pugh A and B).

### 2.3. Afatinib

Phase II/III trials reported a mild rate (10–20%) of any ALT elevations (G3/4: 1.7%), with a 0.2% risk of hepatic failure [35,36,37,38,39,40]. The periodic monitoring of LITs is recommended, and confirmed ALT elevations >5 × ULN should lead to temporary discontinuation, which should become permanent if laboratory values do not improve significantly or resolve within a few weeks, or if symptoms or jaundice arise. Restarting therapy is usually, but not always, followed by recurrence of the serum enzyme elevations. Afatinib hepatotoxicity appears to be less frequent and less severe than with Gefitinib [41,42], and due to the lack of cross-reactivity with other TKIs of EGFR, in some situations, Afatinib may represent an important treatment option when Gefitinib or Erlotinib-induced hepatotoxicity develops [43,44,45,46]. Afatinib has not been studied in patients with severe (Child Pugh C) hepatic impairment, whereas adjustments to the starting doses are not necessary in patients with mild/moderate (Child Pugh A and B) hepatic impairment.

### 2.4. Alectinib

Pre-registration studies reported a mild rate (13–26%) of any ALT elevations (G3/4: 0–5.1%) [47,48,49], similar (8.3–34%, G3/4: 5.7%) to that reported in phase III trials at a dose of 600 mg twice a day. Alectinib therapy was also associated with frequent elevations (47%) in alkaline phosphatase (ALP) and bilirubin (39%), but these abnormalities were usually mild-to-moderate in degree, as well as asymptomatic and transient in nature [50,51,52]. LITs should be performed every two weeks during the first three months of treatment, then once a month and as clinically indicated, with more frequent testing in patients who develop ALT and bilirubin elevations. For ≥G3 hepatotoxicity, the dose should be suspended until toxicity resolves and the value returns to baseline, or <G1 and drug may be resumed at a reduced dose (450 mg twice daily). In case of ALT increase ≥3 × ULN with bilirubin >2 × ULN, Alectinib should be permanently discontinued. There is no evidence of the cross-reactivity of ALK with other TKIs (such as Crizotinib or Ceritinib), and switching to another TKI may be appropriate [53,54]. Increased blood levels of Alectinib occurred in patients with severe hepatic impairment (Child Pugh C), and therefore, the recommended dose in such patients is 450 mg twice daily.

### 2.5. Alpelisib

In Alpelisib and Fulvestrant-treated patients, some degree of ALT elevation was shown in up to 44% (G3/4 in 3.5%). Confirmed ALT elevation >5 × ULN should lead to a dose reduction or temporary interruption. In patients with clinically evident liver injury and/or jaundice, restarting therapy should be done very cautiously, whereas in patients with ALT elevations without jaundice or symptoms, the reintroduction of therapy can be attempted with tight LITs controls. Cross-sensitivity to liver injury is uncommon among this class of TKIs, but there is no clear information or shared adverse event sensitivity of Alpelisib with other TKIs [55,56,57,58].

### 2.6. Avapritinib

Although the rate of all grades of ALT increase was not mentioned in prelicensure and registration studies, a 0.8% risk of liver failure was reported in a phase I dose escalating study [59,60,61]. No dose adjustment is recommended for patients with mild to moderate hepatic impairment, whereas the recommended dose of Avapritinib has not been established for patients with severe hepatic impairment.

### 2.7. Axitinib

Any elevation in serum ALT was reported in up to 22% of patients (G3/4 events occurring in 1%), with no cases of liver failure [62,63,64,65,66,67]. No starting dose adjustment is required in patients with mild hepatic impairment (Child Pugh A). The dose should be reduced by approximately half in patients with baseline moderate hepatic impairment (Child Pugh B), since the blood levels of the drug were shown to be higher in these subjects. Axitinib has not been studied in patients with severe hepatic impairment (Child Pugh C). Weekly monitoring of LITs is recommended, and confirmed serum ALT elevation >5 × ULN should lead to dose reduction or temporary discontinuation. Since a cross-reactivity risk for hepatic injury between Axitinib and other TKIs of this class is not reported, switching to other TKIs in the same class may be appropriate.

### 2.8. Binimetinib + Encorafenib

With this TKIs combination, any elevation in serum ALT was reported in up to 26% of patients (G3/4: 6%), although ALT elevation was generally transient and asymptomatic and there were no reports of liver failure [68,69]. LITs should be monitored before initiation, monthly during treatment and as clinically indicated. For G2 hepatotoxicity, doses should be maintained, but if there is no improvement within two weeks, doses should be suspended until toxicity resolves to baseline or <G1 and the treatment may be resumed at the same dose. With G3/4 hepatotoxicity, the permanent discontinuation of the drug is recommended. There is no evidence of cross-reactivity for hepatic injury between Binimetinib + Encorafenib and other TKIs, such as Dabrafenib + Vermurafenib, and switching to other BRAF/MEK inhibitors may be appropriate.

### 2.9. Bosutinib

In large clinical trials of Bosutinib, serum ALT elevations were common, occurring in up to 59% of recipients (G3/4 hepatotoxicity: 10% to 19%). Most cases of ALT elevations occurred early in treatment, with a median onset time of 30 days (more than 80% of patients experienced their first event within the first three months) and with a median duration of ALT flare of 21 days. These abnormalities were usually asymptomatic, leading to the discontinuation of therapy in approximately 2% of patients [70,71,72,73]. LITs should be monitored before initiation, monthly for the first three months and as clinically indicated. ALT levels >5 × ULN should lead to dose suspension until recovery to ≤2.5 × ULN and the restarting dose should be 400 mg once daily. However, if recovery lasts longer than four weeks, permanent drug discontinuation should be considered. Bilirubin ≥2 × ULN requires Bosutinib discontinuation. In patients with hepatic impairment (Child Pugh A to C), blood levels of Bosutinib are increased compared to healthy controls and a daily dose of 200 mg is recommended.

### 2.10. Brigatinib

This TKI is associated with a mild rate (11–22%) of ALT elevations (G3/4: up to 4%) without liver injury. Brigatinib therapy was also associated with frequent elevations in ALP (15% to 29%), but these elevations were usually asymptomatic, mild-to-moderate in degree and transient [74,75,76]. The product label does not recommend monitoring LITs, but hepatotoxicity ≥G3 should lead to temporary discontinuation, which should be permanent if laboratory values do not improve significantly or resolve within a few weeks, or if symptoms or jaundice arise. In patients with severe hepatic impairment (Child Pugh C), Brigatinib dose should be reduced by approximately 40%.

### 2.11. Cabozantinib

A moderate rate (12–73%) of serum ALT elevations (G3/4: up to 6%) has been reported during treatment with 60 mg once daily, without cases of liver failure. When used in combination with Nivolumab, a higher frequency of G3/4 hepatotoxicity has been reported. Serum ALP elevations were also common and were >3 × ULN in 3% of patients [77,78,79,80,81]. Monitoring LITs before initiation and throughout treatment is recommended in the product label, as well as tighter LITs monitoring when in combination with Nivolumab. Serum ALT elevations >5 × ULN should lead to dose reduction or temporary discontinuation. Cabozantinib dosage should be reduced in patients with moderate hepatic impairment, whereas it should be avoided in those with severe hepatic impairment. There is little information on cross-reactivity for hepatic injury between Cabozantinib and other TKIs of the same class.

### 2.12. Capmatinib

Increased ALT levels occurred in 13% of treated patients (G3/4: 4.4–7%). The median time to ALT increase was 1.4 months (range: 0.5 to 4.1 months) [82,83]. Monitoring LITs is recommended prior to the start and every two weeks during the first three months of treatment, then once a month or as clinically indicated, with more frequent testing in patients who develop increased LITs. G3 ALT increase should lead to temporary discontinuation until recovery to baseline. If ALT recovers to baseline within seven days, Capmatinib can be resumed at the same dose; otherwise, it should be resumed at a reduced dose. Any ALT increase combined with total bilirubin >2 × ULN in the absence of cholestasis or hemolysis required permanent Capmatinib discontinuation.

### 2.13. Ceritinib

ALT elevations occurred in up to 60% of patients (G3/4: 17–31%) [84,85,86,87], and approximately 1% of patients required permanent discontinuation due to hepatotoxicity. Patients should be monitored with LITs once a month and as clinically indicated, with weekly testing in patients who develop ALT elevations. Restarting therapy is usually, but not always, followed by recurrence of aminotransferase elevation. G3 hepatotoxicity should lead to temporary discontinuation until recovery to baseline or <G1. Ceritinib can be resumed with a 150 mg daily dose reduction. Any ALT elevation with total bilirubin elevation >2 × ULN requires permanent drug discontinuation. Despite the fact that hepatotoxicity seems to be a class effect of ALK inhibitors, liver injury appears to be less frequent and less severe with Ceritinib or Alectinib than Crizotinib, and a report of two patients with a successful treatment with Ceritinib after Crizotinib-induced hepatitis has been published [88].

### 2.14. Cobimetinib + Vemurafenib

This TKIs combination is commonly associated with serum ALT elevations occurring in 11% to 25% of patients (G3/4: 5% to 11%) [89,90,91,92,93]. The product label recommends monitoring LITs during treatment, and the first ALT G4 elevation should lead to four weeks’ discontinuation, which should become permanent if laboratory values do not improve to ≤G1. The drug should be resumed at the next lower dosing level. Recurrent G4 hepatotoxicity requires permanent drug discontinuation. Adjustment of the starting dose is not required in patients with mild to moderate hepatic impairment (Child Pugh A and B), whereas in severe hepatic impairment (Child Pugh C), the appropriate dose has not been established.

### 2.15. Copanlisib

The rates of ALT elevations ranged from 23% to 26% (G3/4: 2–4%) in patients without cases of liver injury [94,95]. Since its approval and more general use, no published reports of liver injury with jaundice are available. Although Idelalisib, a small molecule with a similar therapeutic target (PI3K), has been linked to acute liver failure, some of which have been fatal, for Copanlisib, there are no product label recommendations for routine LITs assessment or dosage modifications in case of baseline hepatic impairment or hepatotoxicity development. Moreover, there is no published information on cross-sensitivity to hepatic injury among the different PI3K TKIs.

### 2.16. Crizotinib

Elevations in serum ALT levels occurred in up to 38% of patients (G3/4: 1–3%), leading to early drug discontinuation in 2% to 4% of patients. Serum ALT elevation typically occurs 4 to 12 weeks into treatment, usually without jaundice or ALP elevation. Most cases of liver damage due to Crizotinib have been minimal or asymptomatic and the alteration resolved itself within 1–2 months after stopping the drug. Fatal liver failure has been reported in 0.1% of the cases [96,97]. Routine periodic monitoring of LITs every two weeks for the first two months and monthly thereafter and as clinically indicated, is recommended. ALT elevation >5 × ULN with total bilirubin ≤ 1.5 times ULN should lead to temporary drug discontinuation until recovery to baseline or ≤3 × ULN, and then the drug can be resumed at a reduced dose. ALT elevation >3 × ULN with concurrent total bilirubin elevation >1.5 × ULN requires permanent discontinuation. Patients with liver abnormalities during Crizotinib therapy may tolerate other TKIs, such as Erlotinib or Gefitinib. Crizotinib has not been studied in patients with hepatic impairment, however, since it is extensively metabolized within the liver, and plasma concentrations are expected to increase in case of hepatic impairment, the drug should be used with caution in such patients.

### 2.17. Dabrafenib + Trametinib

This TKIs combination is commonly associated with serum ALT elevations occurring in 35% to 42% of treated patients (G3/4: up to 4%), however, severe liver injury has not been reported [98,99,100,101,102]. Similarly, serum ALP elevations occurred in up to 67% of patients. These abnormalities were largely asymptomatic and fully reversible. Monitoring LITs before starting and during therapy is warranted. Serum ALT elevations >5 × ULN or elevations accompanied by jaundice or liver-related clinical events should lead to temporary cessation and not restart until the LITs abnormalities improve or resolve. Restarting requires careful weekly monitoring. A recommended dosage has not been established for patients with moderate to severe (Child Pugh B, C) hepatic impairment.

### 2.18. Dasatinib

Elevation in serum ALT levels occurred in up to 50% of patients, but was usually mild and self-limiting (G3/4: 1–9%), generally responding to dose adjustment and/or temporary discontinuation. The restarting should be at a lower dose. There have been no published reports of severe liver injury, although other TKIs for chronic myeloid leukemia, i.e., Imatinib, Nilotinib and Ponatinib with a similar therapeutic target (BCR-Abl), have been associated with cases of acute liver injury with jaundice. HBVr in a HBsAg positive patient has been reported with Dasatinib, as well as with Imatinib and Nilotinib [103,104,105,106,107,108,109,110,111,112,113]. Serum ALT elevation >5 × ULN should lead to dose reduction or temporary cessation. Therapy can be restarted, possibly with concurrent prednisone use. In patients with clinically evident liver injury and jaundice, restarting therapy should be done with extreme caution. There does not appear to be cross-reactivity with other TKIs and switching to another drug may be the most appropriate approach.

### 2.19. Dacomitinib

A moderate (23%) rate of transient serum ALT elevation (G3/4: 1%) has been reported without liver injury and, importantly, these rates are lower than those reported with other EGRF inhibitors such as Erlotinib and Gefitinib [114,115]. Serum ALT elevations >5 × ULN should lead to treatment interruption and if the alterations persist, are severe or associated with liver-related symptoms or jaundice, or reoccur on restarting treatment, the drug should be permanently discontinued. Patients with liver abnormalities during Dacomitinib may tolerate treatment with other EGFR inhibitors without recurrence of severe injury. No dose adjustment is recommended in patients with mild or moderate hepatic impairment (Child Pugh A, B) whereas the recommended dose has not been established for patients with severe hepatic impairment (Child Pugh C).

### 2.20. Duvelisib

ALT elevation developed in up to 40% of patients (Grade 3/4: 8%). The median time to onset of ALT elevation was two months, with a median duration of one month. Usually, the ALT elevations resolved spontaneously after drug withholding, and most patients were able to restart treatment without recurrence. While there were no reported cases of liver injury with jaundice, up to 35% of patients discontinued Duvelisib because of ALT elevations [116,117,118,119,120]. LITs should be monitored during treatment because Duvelisib affects B cell function, which may be capable of inducing HBVr, although in published trials, reactivation was not reported. For G2 ALT elevation, the same dose should be maintained while monitoring at least every week until ALT <3 × ULN. For G3 ALT elevation, drug should be suspended and weekly monitoring should be started until ALT <3 × ULN. Duvelisib can be restarted at the same dose (first occurrence) or at a reduced dose (15 mg twice daily) for subsequent occurrence. With G4 ALT elevation, Duvelisib needs to be permanently discontinued. Corticosteroids are often used if the liver injury does not resolve rapidly and maintenance of corticosteroids treatment may help to prevent recurrence of injury when restarting therapy. There is no known cross-sensitivity of hepatic injury among the different TKIs of this class.

### 2.21. Entrectinib

Serum ALT elevations are common during therapy (38%; G3/4: 2.9%), however, there are no reported cases of severe liver injury. The median time of ALT increase was two weeks (range: 1 day to 9.2 months) leading to dose interruptions or reductions in 0.8% of patients [121,122]. The product label recommends monitoring LITs every two weeks during the first month of treatment, then monthly thereafter and as clinically indicated. No dose adjustment is recommended for patients with mild hepatic impairment, whereas Entrectinib has not been studied in patients with moderate and severe hepatic impairment. Serum ALT elevations >5 × ULN should lead to dose interruption until recovery to baseline or <G1 and, if resolution occurs within four weeks, the drug could be resumed at the same dose, or otherwise should be permanently discontinued. For recurrent G3, doses should be resumed at 400 mg, a reduced dose if resolution occurs within four weeks. G4 ALT elevations should lead to dose interruption until recovery to baseline or <G1 and then the drug could be resumed at a reduced dose if resolution occurs within four weeks, or otherwise should be permanently discontinued in case of recurrent G4. In patients with clinically evident liver injury and jaundice, restarting therapy should be done with extreme caution. Cross-sensitivity to liver injury is uncommon among the TKIs, but there is no information or shared adverse event of Entrectinib with other antineoplastic TKIs.

### 2.22. Erdafitinib

In the prelicensure clinical trials, LITs abnormalities were frequent (41%) although usually mild (G3/4: up to 2%), without reports of liver injury or liver-related deaths. Since drug approval and a more widespread use, there have been no reports of liver injury. Regular monitoring of LITs is not specifically recommended during therapy, however G3 ALT elevations should lead to dose interruption until recovery to baseline or <G1, and then the drug can be resumed at a reduced dose, whereas G4 ALT elevation requires permanent drug discontinuation. There is no evidence to suggest cross-reactivity for adverse events between Erdafitinib and other FGFR TKIs [123].

### 2.23. Erlotinib

When combined with chemotherapy, an elevation in serum ALT levels is common (45%) (G3/4: 14%) [124,125,126]. Routine monitoring of LITs before starting and during therapy is recommended. Erlotinib is eliminated by hepatic metabolism and biliary excretion; therefore, patients with any hepatic impairment should be closely monitored during therapy. Serum ALT elevation >5 × ULN should lead to temporary discontinuation, which should be permanent if laboratory values do not improve significantly or resolve within three weeks. Erlotinib should be interrupted if total bilirubin is >3 × ULN. Restarting therapy is usually, but not always, followed by the recurrence of ALT elevations. Since a partial cross-sensitivity for liver injury among similar TKIs appears to exist, switching to another TKI of the same class requires careful monitoring. Patients with acute liver failure due to Erlotinib have been treated with corticosteroids, with uncertain results [44,45,127,128,129,130,131,132,133,134,135,136,137,138,139,140,141,142,143,144,145,146].

### 2.24. Everolimus

Serum ALT elevation occurs in nearly a quarter of patients (G3/4: 1–3%), but the alteration is usually mild, asymptomatic and self-limiting, rarely requiring dose modification or discontinuation, and acute liver injury has not been reported despite its wide scale use. Since exposure to Everolimus was increased in patients with moderate hepatic impairment (Child Pugh B), dose reduction is recommended in such patients. Subsequent dosing should be individualized based on therapeutic drug monitoring. Everolimus has not been studied in patients with severe hepatic impairment (Child Pugh C) and therefore should not be used in this population. Importantly, Everolimus is an immunosuppressive agent and has been associated with HBVr (which can be severe and even fatal) in patients with cancer. Importantly enough, this drug has been associated also with reverse seroconversion, i.e., re-appearance of HBsAg in patients with resolved HBV infection, therefore routine screening for HBsAg, anti HBs and anti-HBc before starting therapy is mandatory [147,148,149,150,151,152,153,154,155,156,157].

### 2.25. Fedratinib

All grades of ALT elevations occurred in 43–53% (G3/4: 1–3%) of patients. The median time to onset of ALT elevation is one month, with 75% of cases occurring within three months. Monitoring LITs at baseline, during treatment and as clinically indicated is recommended. For G3/4 ALT elevation, the drug should be interrupted until resolved to baseline or ≤G1, at which point the drug can be restarted at a dose of 100 mg daily below the last given dose. In cases of the re-occurrence of ALT G3/4 elevation, Fedratinib should be permanently discontinued. The drug has not been evaluated in patients with severe hepatic impairment and (Child Pugh C), and therefore should be avoided in such patients. Cross-sensitivity for liver injury is uncommon among these TKIs class, but there is no information on the shared adverse event between other JAK inhibitors (such as Ruxolitinib) [158,159,160].

### 2.26. Gefitinib

In clinical trials, ALT elevation has been reported in 5 to 55% of the cases (G3/4: 2–27%). Despite the frequency of ALT elevation, severe liver injury was never reported. ALT elevation typically occurs 4 to 12 weeks into treatment, and restarting therapy is usually, but not always, followed by rapid recurrence of ALT elevation. Corticosteroid therapy did not appear to prevent the recurrence. Monitoring LITs during therapy is recommended and the drug should be discontinued in patients with worsening liver function or with severe hepatic impairment. G3/4 ALT elevation requires drug interruption until a reduction to baseline or <G1. The drug can be restarted at 100 mg daily dose below the last given dose. Re-occurrence of a G3/4 ALT elevation requires permanent treatment discontinuation. Patients with liver abnormalities during Gefitinib therapy generally tolerate other TKIs without the recurrence of severe injury [161,162,163,164,165,166,167,168,169,170]. The drug exposure was increased by 40% to 166% in patients with mild to severe hepatic impairment, and therefore, such patients should be monitored during treatment for adverse reactions.

### 2.27. Gilteritinib

Elevation in serum ALT levels is common during therapy, occurring in 42% of patients and rising above 5 × ULN in 14%, but without cases of acute liver injury. ALT elevations >5 × ULN should lead to temporary discontinuation, which should be permanent if laboratory values do not improve significantly or resolve within a few weeks, or if symptoms or jaundice arise [171].

### 2.28. Ibrutinib

In the prelicensure clinical trials, ALT elevation during therapy occurred in 20% to 30%, generally mild and self-limited. In controlled trials, there were no reports of liver injury or a need for early discontinuation because of hepatotoxicity [172,173,174]. Nevertheless, rare cases of acute liver injury, including acute liver failure and severe onset of HBVr, have been reported. Serum ALT elevations >5 × ULN should lead to dose reduction or temporary cessation. In patients with clinically apparent liver injury and jaundice, restarting therapy should be done with caution. Cross-sensitivity to liver injury is uncommon among other BKT TKIs and, in many situations, switching to another one may be appropriate. Importantly, patients who are to receive therapy with Ibrutinib should be screened for HBV markers and, if positive, managed according with HBV prophylaxis guidelines [17,19,20,175].

### 2.29. Idelalisib

The rates of ALT elevations ranged from 35% to 50%. Serum ALT elevations typically arise within 4 to 12 weeks of starting therapy and usually resolved rapidly with temporary discontinuation. Fatal and/or serious hepatotoxicity occurred in up to 14% of treated patients and for this reason, this drug has a warning box for hepatotoxicity, recommending LITs monitoring prior and during treatment. In some instances, when ALT remained high despite stopping therapy, corticosteroids appeared to have a beneficial effect. Due to the effects on B cell function, it may also be capable of inducing HBVr, although in published trials, this event has never been reported. Idelalisib should not be used with other agents with a potential hepatotoxic effect, and regular monitoring of LITs every 2 to 4 weeks is recommended during the first six months and every 1 to 3 months thereafter, with more frequent monitoring if serum ALT values rise. For ALT >3 − 5 × ULN, Idelalisib can be maintained at the same dose with weekly monitoring until ALT < 1 × ULN. The drug should be withheld with ALT >5 × ULN, continuing weekly LITs monitoring until the abnormality is resolved. The treatment may be resumed at a reduced dose (100 mg twice a day). Idelalisib should be permanently discontinued. Corticosteroids are often successfully used for treatment of liver injury that does not resolve rapidly after stopping the TKI. Moreover, continuing the corticosteroids may help to prevent the recurrence of injury when restarting therapy. There is no known cross-sensitivity to hepatic injury among the different TKIs of this class. Patients with any baseline hepatic impairment should be closely monitored for signs of toxicity [32,176,177,178,179,180,181,182,183,184,185,186,187,188,189,190].

### 2.30. Imatinib

Elevation in ALT levels occurred in approximately 20% of treated patients (G3/4: 2–6.8%), requiring treatment discontinuation in <1% of patients. Assessment of LITs before and monthly thereafter, or as clinically indicated, is recommended. Bilirubin >3 × ULN or ALT >5 × ULN requires drug withholding until levels return to <1.5 and <2.5 × ULN, respectively, and then Imatinib can be resumed at a reduced daily dose (i.e., 400 mg to 300 mg, 600 mg to 400 mg or 800 mg to 600 mg daily), depending on the initial severity of the event [111]. Patients with severe hepatic impairment tend to have higher exposure to both Imatinib and its metabolites than patients with normal hepatic function. Therefore, those with mild and moderate hepatic impairment (Child Pugh A, B) do not require a dose adjustment and should be treated with the recommended doses, whereas a 25% dose reduction is recommended for patients with severe hepatic impairment (Child Pugh C).

### 2.31. Infigratinib

In pre-registration trials, ALT elevations arise in 51% (G3/4: 6%) of patients. ALT elevation was generally self-limited and rapidly resolving with or without dose adjustments, and none of the patients developed liver injury or jaundice. Indeed, since its approval, there have been no reports of severe liver injury. Regular monitoring of LITs is not specifically recommended, but in case of ALT elevations LITs monitoring is advisable, due to the risk of liver injury. The recommended dosage of Infigratinib for patients with mild or moderate hepatic impairment is 100 mg or 75 mg once daily for 21 consecutive days, followed by seven days off therapy in 28-day cycles, respectively. In patients with severe hepatic impairment, a recommended dose has not been established. ALT elevations > 5 × ULN or any elevations accompanied by jaundice or symptoms should lead to dose reduction or temporary cessation until the abnormalities resolve, or an alternative cause is identified. There is no evidence to suggest cross-reactivity for adverse events, hypersensitivity or hepatic injury between Infigratinib and other FGFR TKIs [191].

### 2.32. Lapatinib

Hepatotoxicity has been observed in clinical trials and post marketing experience (12–40%; G3/4 up to 13%), and may occur from days to several months after initiation of treatment. This adverse event may be severe and fatalities have been reported, although the causality of deaths is uncertain. For this reason, Lapatinib has a warning box for hepatotoxicity recommending LITs monitoring prior to and every 4 to 6 weeks during treatment, and as clinically indicated thereafter. The reduction or permanent discontinuation of the drug is required in case of severe changes in LITs [192,193]. Patients with severe hepatic impairment (Child Pugh C) should have their dose reduced from 1.250 mg/day to 750 mg/day (HER2 positive metastatic breast cancer indication) or from 1.500 mg/day to 1.000 mg/day (hormone receptor positive, HER2 positive breast cancer indication).

### 2.33. Larotrectinib

ALT increase occurred in up to 45% (G3/4: 11%) of patients. The median time to onset of the alteration was two months (range: 1 month to 13 months) with increased ALT levels leading to dose modifications in 6% and permanent drug discontinuation in 2% of patients, respectively. LITs should be monitored every two weeks during the first month, then monthly thereafter and as clinically indicated. Larotrectinib clearance is reduced in subjects with moderate to severe (Child Pugh B and C) hepatic impairment; therefore, the recommended starting dose should be reduced by 50% in such patients, whereas no dose adjustment is recommended for patients with mild hepatic impairment (Child Pugh A) [194,195,196].

### 2.34. Lenvatinib

Across clinical studies, enrolling patients with malignancies other than hepatocellular carcinoma (HCC), G3/4 ALT flare occurred in 4% of patients, and fatal events, including hepatic failure, acute hepatitis and hepatorenal syndrome, occurred in 0.5% of patients. Among patients treated for HCC, G3/4 ALT flare occurred in 3% of patients, and 2% percent of patients discontinued Lenvatinib. LITs should be monitored at baseline and then every two weeks for the first two months, and at least monthly thereafter during treatment, whereas patients with HCC should be closely monitored for signs of liver failure. G3/4 ALT elevations should lead to a dose interruption until improvement to baseline or <G1 ALT levels, and then the drug could be resumed at a reduced dose or discontinued, depending on the severity and persistence of hepatotoxicity. G4 ALT elevation and hepatic failure requires permanent drug discontinuation [197,198].

### 2.35. Lorlatinib

Increased ALT of any grade occurred in up to 28% of patients (G3/4: 1–2.1%), usually within three days, and returned to normal limits after a median of 15 days (7 to 34 days). This drug is contraindicated in patients treated with both moderate and strong CYP3A inducers, which should be discontinued prior to initiating Lorlatinib. If concomitant use of moderate CYP3A inducers cannot be avoided, LITs should be monitored 48 h after initiating Lorlatinib and at least thrice during the first week of treatment. Monitoring LITs is recommended at baseline and during treatment. ALT elevations >5 × ULN should lead to dose interruption and if the alterations persist, are severe or reoccur upon restarting, Lorlatinib should be permanently discontinued. Patients developing liver injury under treatment with a specific ALK inhibitor can often be treated with other ALK TKI without recurrence of liver injury, but careful monitoring is necessary during treatment after hepatotoxicity from a related TKI agent [199]. No dose adjustment is recommended for patients with mild hepatic impairment (Child Pugh A), whereas the recommended dose has not been established for patients with moderate or severe hepatic impairment.

### 2.36. Midostaurin

Elevation in serum ALT levels is common during therapy, occurring in up to 71% of patients (G3/4: 4–20). Hyperbilirubinemia was also reported to occur commonly, however liver injury with jaundice, severe hepatoxicity and deaths from hepatic failure have not been reported. It must be noted that, because of the limited clinical experience with the use of Midostaurin and other FLT3 TKIs, their potential for causing liver injury is not well defined as yet. Serum ALT elevations >5 × ULN should lead to temporary discontinuation, which should be permanent if laboratory values do not improve significantly or resolve within a few weeks, or if symptoms or jaundice arise [200].

### 2.37. Mobocertinib

Increased ALT of any grade occurred in up to 22% of patients (G3/4: 1–2.7%). No LITs monitoring is recommended. Intolerable or recurrent G2 or G3 hepatotoxicity should lead to temporary discontinuation until reaching levels ≤G1, when the drug can be resumed at either the same dose or the next lower dose. G4 toxicity should lead to temporary discontinuation until ≤G1, and the drug can be resumed at the next lower dose if recovery occurs within two weeks, or permanently discontinued if recovery does not occur within two weeks as well as in case of liver toxicity recurrence. No dose adjustment is recommended for patients with mild or moderate hepatic impairment (Child Pugh A, B) whereas the recommended dosage has not been established for patients with severe hepatic impairment (Child Pugh C) [201,202].

### 2.38. Neratinib

Elevation in serum ALT levels was uncommon during therapy, occurring in up to 13% of patients, (G3/4: 1–5%), and was typically mild, self-limited and not associated with symptoms or jaundice. Drug discontinuation due to hepatotoxicity was reported in 1.7% of patients and this side effect may be a class effect among TKI of HER2, although both the frequency and severity vary among different agents. Monitoring LITs is recommended before treatment, monthly for the first three months and every three months thereafter or as clinically indicated. Serum ALT elevations >5 × ULN should lead to temporary discontinuation, which should be permanent if laboratory values worsen or do not resolve or improve significantly within a few weeks, or if clinical symptoms or jaundice arise. Therapy can be restarted at the next lower dose if recovery to ≤G1 is achieved and it is usually, but not always, followed by the recurrence of ALT elevations. G4 ALT elevation requires permanent drug discontinuation [203,204].

### 2.39. Nilotinib

Elevation in serum ALT levels is reported in up to 24% of patients (G3/4: 3–9%), but these abnormalities are usually asymptomatic. There has been only a single published case report of liver injury attributed to Nilotinib. Most other TKIs have been linked to rare instances of clinically evident liver injury, usually arising 1 to 8 weeks into treatment. Monitoring of LITs is recommended at baseline and monthly or as clinically indicated during treatment. ALT levels >5 × ULN should lead to dose reduction or temporary discontinuation. Cross-reactivity of the hepatic injury with other TKI is uncommon, but can occur, therefore drug switching needs to be carefully evaluated [205,206].

### 2.40. Osimertinib

Elevation in serum ALT levels is uncommon (5%; G3/4: 1%). In pre-registration trials, one single evident case of liver injury was reported, however both the clinical features and the causality with Osimertinib were not clearly defined. Since its approval and more widespread use, there have been no reported cases of liver injury. Routine monitoring of LITs is not recommended, but ALT >5 × ULN should lead to discontinuation for up to three weeks. If the level drops to G0-2, the drug can be resumed at 80 mg or 40 mg daily whereas if no improvement is reached within three weeks, Osimertinib should be permanently discontinued. There does not appear to be cross-reactivity with other EGFR inhibitors and, when needed, switching to another TKI may be appropriate [207].

### 2.41. Palbociclib

In large clinical trials, serum ALT elevations occurred in up to 43% of patients (G3/4: 2%), and since its approval and widespread use, there have been several reports of ALT elevation arising after two or three cycles. Aminotransferase levels generally improved after discontinuation but recurred rapidly when Palbociclib was restarted. Serum ALT >5 × ULN or any elevations accompanied by jaundice or clinical symptoms should lead to dose reduction or temporary discontinuation. No dose adjustment is required in patients with mild or moderate hepatic impairment (Child Pugh A and B), whereas for patients with severe hepatic impairment (Child Pugh C), the recommended dose is 75 mg once daily for 21 consecutive days, followed by seven days off treatment to complete a full cycle of 28 days. There is no evidence to suggest cross-reactivity for adverse events, hypersensitivity or hepatic injury between Palbociclib and other CDK inhibitors [208].

### 2.42. Pazopanib

In large clinical trials, serum ALT elevation occurred in up to 60% of patients (G3/4: 9–17%), with total serum bilirubin elevation in approximately one-third. In preliminary trials in different solid tumors, there were rare reports of hepatitis with jaundice (<1%), but subsequent reports showed fatal instances of liver injury. This drug has a warning box for hepatotoxicity recommending to monitor LITs before initiation of treatment and at least once every four weeks for the first four months, or as clinically indicated. Patients with ALT elevation >3 − 8 × ULN may be maintained on treatment with the weekly monitoring of LITs until ALT levels return to G1 or baseline. In patients with isolated ALT elevations >8 × ULN, the drug should be withheld until they return to G1 or baseline; if the potential benefit for reinitiating treatment outweighs the risk of hepatotoxicity, the drug could be reintroduced at a reduced dose of no more than 400 mg once daily, maintaining LITs monitored weekly for eight weeks. Following the reintroduction of Pazopanib, a recurrent ALT increase >3 × ULN requires drug discontinuation. The dosage of Pazopanib in patients with moderate hepatic impairment (Child Pugh B) should be reduced to 200 mg per day, whereas there are no data for patients with severe hepatic impairment (Child Pugh C); therefore, the use of Pazopanib is not recommended in such patients [209,210,211,212,213].

### 2.43. Pemigatinib

In pre-registration clinical trials, ALT elevation was reported in 43% of patients (G3/4: 4.1%). The elevation was typically self-limited and resolved rapidly with or without dose adjustments. Since its approval, there have been no reports of clinically evident severe liver injury. Elevations in serum bilirubin were also common, but usually in the context of cholangiocarcinoma with partial or complete biliary obstruction. The recommended drug dose for patients with severe hepatic impairment (Child Pugh C) is 9 mg within the specific schedule (intermittent or continuous), designed for the specific indication [214].

### 2.44. Pexidartinib

Elevation in serum ALT levels is common, occurring in 50% to 90% of patients (G3/4: 12–20%). In addition, elevation in ALP levels occur in up to 20% of patients. In registration trials, liver injury with jaundice developed in 5% of patients. The time of onset of liver injury was usually between 2 and 6 weeks, and the pattern of liver enzyme elevations was either mixed or solely cholestatic. Liver biopsy documented both bile duct injury and loss, and three patients under treatment for conditions other than Tenosynovial giant cell tumors, developed bile duct paucity and features of vanishing bile duct syndrome that ultimately led to liver transplantation in one subject. For this reason, this drug has a warning box for hepatotoxicity, recommending monitoring of LITs before initiation of treatment, weekly for the first eight weeks, every two weeks for the next month and every three months thereafter. Patients with ALT elevations >3 − 5 × ULN should discontinue the treatment with a weekly monitoring of LITs until returning to G1 or baseline; if this occurs within four weeks from discontinuation, the drug can be resumed at a reduced dose, otherwise Pexidartinib needs to be permanently discontinued. For patients with ALT >5 − 10 × ULN, treatment should be interrupted and LITs should be checked twice weekly until they return to G1 or baseline; if this happens within four weeks, the drug could be resumed at a reduced dose, otherwise Pexidartinib needs to be permanently discontinued. In patients with ALT >10 × ULN, the drug should be discontinued permanently. Rechallenge with a reduced dose of Pexidartinib may result in a recurrence of increased ALT, bilirubin or ALP. Pexidartinib should be avoided in patients with pre-existing increased serum ALT, total bilirubin or direct bilirubin >ULN, or active liver or biliary tract disease, including increased ALP [215,216,217,218].

### 2.45. Ponatinib

In large clinical trials, elevation in ALT levels occurred in up to 56% of patients (G3/4: 8%). While these abnormalities were reversible in most patients, they were prolonged or severe in many others. Episodes of clinically evident progressive hepatic failure and death were reported in clinical trials. This drug has a warning box for hepatotoxicity, which recommends monitoring LITs before initiation of treatment and at least monthly or as clinically indicated. A ≥G2 ALT elevation occurring at 45 mg initial dose requires drug interruption until ≤G1, and a new start at 30 mg after recovery. When the starting dose is 30 mg, drug interruption is required until ≤G1 and a new start at 15 mg after recovery; when starting dose is 15 mg, permanent drug discontinuation is required. Hepatotoxicity ≥G2 with bilirubin >2 × ULN requires permanent treatment discontinuation. Ponatinib is metabolized in the liver largely through the CYP 3A4 pathway, and liver injury may be related to the production of a toxic intermediate. Because of this metabolic pathway, Ponatinib is susceptible to drug-drug interactions (DDI) when using agents that induce or inhibit CYP 3A4. As hepatic elimination is a major route of excretion for Ponatinib, hepatic impairment may result in increased drug exposure and increased risk for adverse reactions; therefore, in patients with moderate to severe (Child Pugh B or C) hepatic impairment, Ponatinib should be avoided unless the benefit outweighs the possible risk of overexposure [219,220].

### 2.46. Pralsetinib

ALT increase occurred in up to 46% of patients (G3/4: up to 6%). The median time to first onset was 22 days (range: 7 days to 1.7 years). LITs should be monitored prior to initiation, every two weeks during the first three months, then monthly thereafter and as clinically indicated. G3 or G4 toxicity requires the withholding of Pralsetinib with once-a-week ALT monitoring until resolution to baseline or G1; then, the drug can be resumed at a reduced dose. If hepatotoxicity recurs at G3 or greater, the drug needs to be permanently discontinued. Pralsetinib has not been studied in patients with moderate to severe hepatic impairment (Child Pugh B, C), whereas no dose adjustment is required for patients with mild hepatic impairment (Child Pugh A) [221,222].

### 2.47. Regorafenib

Severe and sometimes fatal hepatotoxicity events have been observed in clinical trials; therefore, this drug has a warning box for hepatotoxicity and recommends monitoring LITs before initiation of treatment and at least every two weeks during the first two months of treatment, or more frequently as clinically indicated. No clinically important differences in the mean exposure of Regorafenib or the active metabolites M-2 and M-5 were observed in patients with HCC and mild/moderate hepatic impairment (Child Pugh A and B), compared to patients with normal hepatic function, therefore no dose adjustment is recommended in such patients. Regorafenib is not recommended in patients with severe hepatic impairment (Child Pugh C) as it has not been studied in this population [223,224].

### 2.48. Ribociclib

In preregistration and large clinical trials, ALT elevation occurred in 46% of patients (G3/4: 9–14%). LITs should be evaluated initiating treatment and then every two weeks for the first two cycles, at the beginning of the subsequent four cycles and as clinically indicated. Ribociclib is extensively metabolized in the liver, largely through the CYP 3A4 pathway, and liver injury might be induced by the production of a toxic or immunogenic intermediate. On the other hand, the inhibition of CDK4/6 may also affect hepatocytes and cause direct hepatotoxicity. G3 ALT elevation requires dose interruption until recovery to baseline with a possible drug restart at the next lower dose level. G3 recurrence or G4 ALT elevation requires permanent treatment discontinuation. No dose adjustment is necessary in patients with mild hepatic impairment (Child Pugh A), whereas a reduced starting dose of 400 mg is recommended in patients with moderate and severe hepatic impairment (Child Pugh B or C) [225,226].

### 2.49. Ripretinib

ALT increase occurred in 12% of patients (G3/4: 1.2%). Monitoring LITs, as well as dose adjustment in patients with mild hepatic impairment (Child Pugh A), is not required. A recommended dosage and specific monitoring protocol have not been established for patients with moderate or severe hepatic impairment (Child Pugh B, C) [227,228].

### 2.50. Ruxolitinib

ALT elevation occurred in 25–38% of patients (G3/4: 1%) and were generally self-limited, asymptomatic and mild, with no cases of clinically evident liver injury. Importantly, there have been several published reports of HBVr, therefore patients undergoing Ruxolitinib should be screened for HBV markers and managed according to HBV reactivation guidelines (21). Ruxolitinib is metabolized in the liver largely through the CYP 3A4 pathway and therefore is susceptible to DDI agents that inhibit or induce this cytochrome activity. Blood levels of Ruxolitinib increase in mild to severe hepatic impairment (Child Pugh A to C), and therefore dose reduction is recommended in such patients [229,230].

### 2.51. Selpercatinib

Increased ALT occurred in 56% of patients (G3/4: 12%). The median time to onset of ALT elevation was 5.8 weeks (range: 1 day to 2.5 years). Monitoring LITs prior to initiation and every two weeks during the first three months, then monthly thereafter and as clinically indicated, is recommended. G3/4 ALT elevation requires dose interruption with LITs monitoring once weekly until resolution to baseline or G1; the drug can be restarted at two-dose levels reduction with LITs monitoring once weekly. Dosage can be increased by one dose after a minimum of two weeks without recurrence, and then further increased prior to the hepatotoxicity after a minimum of four weeks without recurrence. The Selpercatinib dose should be reduced when administered to patients with severe hepatic impairment (Child Pugh C), whereas no modification is recommended for patients with mild or moderate hepatic impairment [231,232].

### 2.52. Sorafenib

Sorafenib-induced liver dysfunction has been reported in 11% of patients (G3/4: 3%), although liver injury has been reported only in 0.06% of treated subjects. Monitoring LITs is recommended and in case of a significant increase of ALT without alternative explanation (such as viral hepatitis or progression of the underlying liver malignancy), treatment should be discontinued [233,234].

### 2.53. Sunitinib

In large clinical trials, ALT elevation was common, occurring in 39–61% of patients (G3/4: 2–5%), and in clinical trials and post-marketing experiences, the treatment has been associated with liver failure or death (0.3%). Therefore, this drug has a warning box for hepatotoxicity; monitoring LITs is recommended before initiation, during each cycle of treatment, and as clinically indicated. Sunitinib should be interrupted for G3/4 drug-related hepatic adverse events and discontinued if there is no resolution, a further increase of LITs or signs and symptoms of liver failure. Sunitinib has also been reported to cause hyperammonemia and encephalopathy with confusion and irritability, even with minimal elevation in serum enzymes and bilirubin and marked increases (4 − 10 × ULN) in serum ammonia levels. Recovery is rapid once Sunitinib is stopped and the syndrome can recur with re-exposure. Interestingly, there appears to be little cross-reactivity of this complication with other same class TKIs. The clinical features of the reported cases of severe acute liver injury due to Sunitinib have suggested a possible mechanism of ischemic damage related to hypotension and anoxia rather than direct hepatic injury.

No dose adjustment is required in subjects with mild or moderate hepatic impairment (Child Pugh A and B), whereas no data exist in subjects with severe hepatic impairment (Child Pugh C) since most studies have excluded patients with significant underlying liver disease at baseline [235,236,237,238].

### 2.54. Temsirolimus

Serum ALT elevation occurred in 30% to 40% of patients (G3/4: 1–3%), but the abnormalities are usually mild, asymptomatic and self-limited, rarely requiring dose modification or discontinuation. Since approval and widespread clinical use, there have been no case reports of severe liver injury. Although to date there have been no reports of HBVr, since Temsirolimus is an immunosuppressive agent, HBVr should be considered a possible complication of this drug. Concentrations of Temsirolimus as well as its metabolite Sirolimus are increased in patients with elevated ALT or bilirubin levels, and therefore, its use is contraindicated in patients with bilirubin >1.5 × ULN, and the dose should be reduced in patients with mild hepatic impairment (Child Pugh A) [239,240].

### 2.55. Tepotinib

Increased ALT occurred in 13% of patients (G3/4: 4.2%) with one reported fatal adverse reaction of hepatic failure (0.2%). The median time to onset of ≥G3 ALT increase was 30 days (range 1 to 178). LITs should be monitored prior to the start of treatment, every two weeks during the first three months and then once a month or as clinically indicated, with more frequent testing in patients who develop increased ALT or bilirubin. G3 hepatotoxicity requires dose interruption until recovery to baseline. If hepatotoxicity recovers to baseline within seven days, Tepotinib can be resumed at the same dose. Otherwise, it should be resumed at a reduced dose. G4 ALT increase or bilirubin >2 × ULN requires permanent drug discontinuation. No dose modification is recommended in patients with mild or moderate hepatic impairment (Child Pugh A and B), whereas the pharmacokinetics and safety of Tepotinib in patients with severe hepatic impairment (Child Pugh C) have not been studied [241].

### 2.56. Tivozanib

Increased ALT occurred in 30% of patients (G3/4: 4%). No dose modification is recommended for patients with mild hepatic impairment (Child Pugh A), but should be reduced in patients with moderate hepatic impairment (Child B), whereas the recommended dosage in patients with severe hepatic impairment (Child Pugh C) has not been established [242].

### 2.57. Tucatinib

Increased ALT occurred in 46% of patients (G3/4: 8%). LITs should be monitored prior to starting, every three weeks during treatment and as clinically indicated. G3 ALT increase or bilirubin >3 − 10 × ULN requires dose interruption until recovery to ≤G1, and then the drug could be resumed at the next lower dose level. G4 ALT increase or bilirubin >10 × ULN requires permanent drug discontinuation. Tucatinib exposure is increased in patients with severe hepatic impairment (Child Pugh C), therefore requiring a reduced dose. No dose adjustment is required for patients with mild or moderate hepatic impairment (Child Pugh A and B) [243].

### 2.58. Vandetanib

In large clinical trials, increased ALT occurred in 51% of patients (G3/4: 2%), without reports of severe liver injury or hepatic failure. Since approval and a wider scale use, there have been no published reports of hepatotoxicity, and the product label does not include discussion of hepatotoxicity. There are limited data in patients with liver dysfunction, however the use of Vandetanib is not recommended in patients with moderate and severe hepatic impairment (Child Pugh Band C), as safety and efficacy have not been established [244].

### 2.59. Zanobrutinib

Increased ALT occurred in 28% of patients (G3/4: 0.9%) without reports of liver injury or liver-related deaths. Nonetheless, other Bruton’s kinase inhibitors (Ibrutinib and Acalabrutinib) have been associated with rare cases of acute liver injury, including acute liver failure as well as cases of HBVr. No dosage modification is recommended in patients with mild to moderate hepatic impairment (Child Pugh A and B). Although Zanobrutinib has not been fully evaluated in patients with severe hepatic impairment (Child Pugh C), in such patients the recommended dosage should be reduced [245].

## 3. Discussion

The introduction of small-molecule PKIs in clinical oncology has dramatically improved the prognosis of certain types of cancers and in the last decades, the number of newly approved molecules has rapidly increased. Unfortunately, hepatotoxicity is a major safety concern of these drugs, with many of them having been implicated in cases of clinically evident liver injury and nearly 10% of the 62 approved compounds containing a drug labeling warning for hepatotoxicity. An interesting point, although not addressed by our review, is the issue of the combined use of a natural agent together with PKI. Data from the literature hypothesize that traditional Chinese medicinal herbs might increase efficacy while reducing toxicity if administered in combination with PKI, i.e., EGFR-TKI for Advanced Non-Small-Cell Lung Cancer [246]. However, only scattered evidence on this topic has been documented outside of China, and most of their reports do not specifically refer to liver toxicity. More importantly, all these evidences require further verification by well-designed studies. Above all, we should also highlight the fact that among the vast market of “natural products”, in certain cases, not all the components are clearly declared; this can prevent physicians from properly avoiding potentially major interferences with the CYP 3A4 pathway (with an inhibition or induction of this specific hepatic microsomal activity), leading to the production of several potentially toxic or immunogenic intermediates.

Moreover, several PKIs have also been implicated in causing HBV reactivation, both in patients with overt and resolved infection. Indeed, some PKIs act as immunosuppressants, lowering the normal immune surveillance pressure against HBV and enhancing HBV viral replication, which is responsible for the rapid spreading of the infection to most of the hepatocytes, followed by hepatitis flare when the host immunity recovers after PKI withdrawal. Some PKIs, even if not specifically identified as immunosuppressant agents, may induce HBVr after a tumor response, supporting the hypothesis of immune restoration leading to HBVr [11,12,247]. Overall, the severity and consequences of the side effects due to either HBVr or drug-related hepatotoxicity is driven by the pre-treatment stage of the liver function, extent of liver damage, patient’s age and performing status.

## 4. Future Directions

Despite the progress of PKIs for cancer treatment, the safety issues are often the main reason for their reduced effectiveness in the real practice since toxicity (and particularly hepatotoxicity) may cause their temporary or definitive discontinuation. For this reason, all the research efforts must aim to maximize the development of safer PKIs that can be defined not only in the pre-clinical and clinical phases, but also in the post-marketing phase. Research should focus on the identification of possible genetic predictors of hepatic metabolism and bio-transformation of the drugs, so that the therapeutic choice on the type and dose of the PKI should be tailored for each individual patient by identifying the one presenting both maximum efficacy and safety. Further efforts should be made in understanding the real rates of hepatotoxicity in cancer patients, overcoming all the confounding factors. Indeed, these patients may have already been treated with chemotherapies that could have impaired their liver function, or they may have metastatic liver involvement or may be on concomitant treatment with other drugs that could be either responsible for DDIs with PKIs or being primarily hepatotoxic themselves. Furthermore, as most of the data on hepatotoxicity comes from pre-clinical and clinical studies on fairly selected patients, and the post-marketing data are often based only on the most severe described cases, more information from the real practice with large-scale studies and possible long-term follow-ups are mostly needed. The aim should be to carefully assess the occurrence of liver injury or even liver failure. More data are needed in patients with pre-existing liver damage who need PKI for tumors arising in organs different from the liver, but above all, for those who need PKI for HCC or other tumors in the context of a pre-existing or even advanced liver disease. More information is also needed on the cross-reactivity of risk for hepatic injury and the possibility of safe therapeutic switches between different PKIs in order to provide each patient with the most effective treatment strategies.

The increased access of patients to these therapies and the risk of liver damage requires greater synergy and collaboration between oncologists and hepatologists, both in the correct definition of the patient at greater risk of adverse events before starting PKI, but also in the early identification of patients who develop liver damage for the most appropriate medical management of this complication. Although it is not easy to predict and treat the progress of most drug-related liver damages, it is essential, not only to ensure a prompt recovery, but mainly to prevent the evolution towards liver failure, as cancer patients currently do not have the opportunity for liver transplantation. Finally, the choice regarding the continuation of the PKI treatment could be shared between the oncologist and hepatologist on a case-by-case level and tailored to the risk/benefit ratio, especially for patients who are prone to respond to anticancer therapy.

## 5. Conclusions

The issue of potential drug hepatotoxicity and HBVr has always represented a major concern when treating cancer patients, although the current PKIs seem to be less dangerous than the previous standard chemotherapies [248]. Certainly, the occurrence of hepatotoxicity impacts both patient care and the survival rate since it deeply influences the management of a crucial oncological treatment by imposing either suspension or permanent discontinuation, or switching to a different drug. Therefore, any treatment choice after PKI-hepatotoxicity must take into account the potential beneficial risks while preventing any further liver deterioration. To minimize the consequences of this event, it is essential to:

Carefully select patients suitable for a specific PKI:

- Adapt the recommended PKI dose according to the pre-existing hepatic impairment;
- Avoid DDIs, also informing patients about the potential risks of concomitant use of over-the-counter drugs which could alter the metabolism of PKIs, thus favoring the formation of potentially hepatotoxic metabolites;
- Set and maintain the recommended monitoring protocol for LITs.

Finally, oncologists must be aware that is possible to avoid the use of a specific PKI when the risk of liver toxicity exceeds the possible benefits. In addition, they must be ready to promptly reduce or suspend the drug in the event of any LITs alteration, referring to hepatologists for an additional assessment of all the patients with a baseline hepatic dysfunction prior to starting PKI, or those developing liver damage during treatment for an appropriate medical management.

## Figures and Tables

**Table 1:** Main factors involved in liver injury secondary to PKIs approved for cancer.

Drug	Primary Targets	Indications	Any ALT Elevation (%)	G3/4 ALT Elevation (%)	Liver Failure (%)	Drug Labeling Warnings for Liver Injury	Drug Labeling Boxed Warnings for Liver Injury
Abemaciclib	CDK4/6	In combination with an aromatase inhibitor or with fulvestrant or as a monotherapy for breast cancers	30–48	4.6–7	0	Yes	No
Acalabrutinib	BTK	MCL, CLL, SLL	4.5–20	1–1.9	0	No	No
Afatinib	EGFR, HER2, ErB4	NSCLC	10–20	1.7	0.2	Yes	No
Alectinib	ALK, RET	ALK-positive NSCLC	8.3–34	0–5.7	0	Yes	No
Alpelisib	PIK3	In combination with fulvestrant for breast cancer	11–44	0–3.5	0	Yes	No
Avapritinib	PDGFRα	GIST harboring PDGFRA exon 18 mutation, and PDGFRA D842V mutations	na	na	0.8	No	No
Axitinib	VEGFR1/2/3	RCC	22	1	0	Yes	No
Binimetinib plus Encorafenib	MEK1/2	BRAFV600E/K melanoma	7–26	6	0	Yes	No
Bosutinib	BCR-Abl	CML	32–59	10–19	0	Yes	No
Brigatinib	ALK	ALK-positive NSCLC	11–22	1.5–4	0	No	No
Cabozantinib	RET, VEGFR2	Medullary thyroid cancers, RCC, HCC	12–73	0–6	0	Yes	No
Capmatinib	c-MET	NSCLC with MET exon 14 skipping	13	4.4–7	0	Yes	No
Ceritinib	ALK	ALK-positive NSCLC resistant to crizotinib	35–60	17–31	0	Yes	No
Cobimetinib plus Vemurafenib	MEK1/2 and B-Raf	BRAFV600E/K melanomas	11–25	5–11	0	Yes	No
Copanlisib	PI3K	Relapsed follicular lymphoma	23–26	2–4	0	No	No
Crizotinib	ALK, ROS1	ALK or ROS1-postive NSCLC	10–38	1–3	0.1	Yes	No
Dabrafenib plus Trametinib	B-Raf, MEK 1/2	BRAFV600E/K melanomas	35–42	0–4	0	No	No
Dasatinib	BCR-Abl	CML positive for the Philadelphia chromosome	50	1–9	0	No	No
Dacomitinib	EGFG	EGFR-mutant NSCLC	23	1	0	No	No
Duvelisb	PI3K	Relapsed or refractory CLL, SLL, FL	31–40	3–8	0	No	No
Entrectinib	NTRK, ROS1	Adult with NSCLC and adult and pediatric patients with NTRK gene fusion solid tumors	38	2.9	0	Yes	No
Erdafitinib	FGFR1/2/3/4	Urothelial bladder cancers	41	1–2	0	No	No
Erlotinib	EGFR	NSCLC, pancreatic cancers	45	14	0	Yes	No
Everolimus	FKBP12/mTOR	HER2-negative breast cancers, pancreatic neuroendocrine tumors, RCC, angiomyolipoma’s, subependymal giant cell astrocytoma	21	1–3	0	No	No
Fedratinib	JAK-2, FLT3	Intermediate or high-risk, primary or secondary Myelofibrosis	43–53	1–3	0	Yes	No
Gefitinib	EGFR	NSCLC	5–55	2–27	0	Yes	No
Gilteritinib	Flt3	AML	42	14	0	No	No
Ibrutinib	BTK	CLL, mantle cell lymphomas, marginal zone lymphomas	20–30	NA	0	No	No
Idelalisib	PI3K-delta	Relapsed CLL in combination with rituximab, relapsed FL and relapsed SLL in patients who have received at least two prior systemic therapies	35–50	8–14	14	Yes	Yes
Imatinib	BCR-Abl	Ph+ CML or ALL, aggressive systemic mastocytosis, chronic eosinophilic leukemias, dermatofibrosarcoma protuberans, hyper eosinophilic syndrome, GIST, myelodysplastic/myeloproliferative disease	20	2–6.8	0	Yes	Yes
Infigratinib	FGFR2	Cholangiocarcinomas with FGFR2 fusion proteins	51	6	0	No	No
Lapatinib	EGFR, ErbB2/HER2	In combination with capecitabine or letrozole for advanced or metastatic breast cancer	12–40	2–13	0	Yes	Yes
Larotrectinib	TRKA/B/C	Solid tumors with NTRK fusion proteins	25–45	0–11	0	Yes	No
Lenvatinib	VEGFR, RET	Refractory differentiated thyroid cancer, in combination with Pembrolizumab or Everolimus for RCC; unresectable HCC; in combination with Pembrolizumab for advanced endometrial carcinoma	6–11	1–4	0.5	Yes	No
Lorlatinib	ALK	ALK-positive metastatic NSCLC	9–28	1–2.1	0	Yes	No
Midostaurin	FLT3	AML FLT3 mutation positive, ASM, SM-AHN, MCL	31–71	4–20	0	No	No
Mobocertinib	EGFR	NSCLC with EGFR-positive exon 21 o 20 insertions whose disease has progressed on or after platinum-based chemotherapy	8–22	1–2.7	0	No	No
Neratinib	ErbB2/HER2	HER2-overexpressed/amplified breast cancer, to follow adjuvant trastuzumab-based therapy	9–13	1–5	0	Yes	No
Nilotinib	BC-Abl	Ph + CML	4–24	3–9	0	Yes	No
Osimertinib	EGFR T970M	EGFR T790M mutation positive NSCLC	5	1	0	No	No
Palbociclib	CDK4/6	Estrogen receptor- and HER2-positive breast cancers	36–43	2	0	No	No
Pazopanib	VEGFR1/2/3	RCC, soft tissue sarcomas	10–60	9–17	0	Yes	Yes
Pemigatinib	FGFR2	Advanced cholangiocarcinoma with an FGFR2 fusion or rearrangement	43	4.1	0	No	No
Pexidartinib	CSF1R	Tenosynovial giant cell tumors	50–90	12–20	5	Yes	Yes
Ponatinib	BCR-Abl	Ph + CML or ALL	56	8	0	Yes	Yes
Pralsetinib	RET	RET-fusion, NSCLC, medullary thyroid cancer	23–46	1–6	0	Yes	No
Regorafenib	VEGFR1/2/3	Colorectal cancers, HCC	45	6	0.3–1.6	Yes	Yes
Ribociclib	CDK4/6	Combination therapy with an aromatase inhibitor for breast cancers	46	9–14	0	Yes	Yes
Ripretinib	Kit, PDGFRα	GIST	12	1.2	0	No	No
Ruxolitinib	JAK1/2/3, Tyk	Myelofibrosis, polycythemia vera	25–38	1	0	No	No
Selpercatinib	RET	NSCLC and thyroid cancers	56	12	0	Yes	No
Sorafenib	VEGFR1/2/3	HCC, RCC, differentiated thyroid cancer	11	3	0.06	Yes	No
Sunitinib	VEGFR2	GIST, pancreatic neuroendocrine tumors, RCC	39–61	2–5	0.3	Yes	Yes
Temsirolimus	FKBP12/mTOR	RCC	30–40	1–3	0	Yes	No
Tepotinib	MET	NSCLC with MET mutations	13	4.2	0.2	Yes	No
Tivozanib	VEGFR2	Third-line treatment of RCC	30	4	0	No	No
Tucatinib	ErbB2/HER2	In combination with trastuzumab and capecitabine for advanced unresectable or metastatic HER2-positive breast cancer	46	8	0	Yes	No
Vandetanib	VEGFR2	Medullary thyroid cancers	51	2	0	No	No
Zanubrutinib	BTK	Mantle cell lymphomas	28	0.9	0	No	No

Mast Cell Leukemia: MCL; Chronic Lymphocytic Leukemia: CLL; Small Lymphocytic Lymphomas: SLL; Non-Small Cell Lung Cancer: NSCLC; Gastrointestinal Stromal Tumor: GIST; Renal Cell Carcinoma: RCC; Chronic Myeloid Leukemia: CML; Hepatocellular Carcinoma: HCC; Follicular Lymphoma: FL; Acute Myeloid Leukemia: AML; Acute Lymphoblastic Leukemia: ALL; Aggressive Systemic Mastocytosis: ASM; Systemic Mastocytosis with Associated Hematological Neoplasm: SM-AHN; Mantle Cell Lymphoma: MCL; not available: NA; Grade 3: alanine aminotransferase (ALT) >5 − 20 × ULN (upper limit of normal) without increase in total bilirubin >2 × ULN; Grade 4: ALT > 20 × ULN.
